# Ursolic Acid Regulates Cell Cycle and Proliferation in Colon Adenocarcinoma by Suppressing Cyclin B1

**DOI:** 10.3389/fphar.2020.622212

**Published:** 2021-01-19

**Authors:** Minhui Yang, Changxiao Hu, Yibo Cao, Wanling Liang, Xiangdong Yang, Tianbao Xiao

**Affiliations:** ^1^ College of Clinical Medicine, Guizhou University of Traditional Chinese Medicine, Guiyang, China; ^2^ Colorectal and Anal Surgery, the First Affiliated Hospital of Guizhou University of Traditional Chinese Medicine, Guiyang, China; ^3^ Colorectal and Anal Surgery, Chengdu Anorectal Hospital, Chengdu, China

**Keywords:** colon adenocarcinoma, ursolic acid, cyclin B1, cell cycle, Proliferation

## Abstract

**Aims:** The biological functions of cyclin B1 (CCNB1) in colon adenocarcinoma (COAD) will be explored in this study. Furthermore, the therapeutic effects and potential molecular mechanisms of ursolic acid (UA) in COAD cells will also be investigated *in vitro*.

**Methods:** COAD data were obtained from Gene Expression Omnibus (GEO) and The Cancer Genome Atlas (TCGA) databases. Differentially expressed genes (DEGs) were determined with differential analysis. The biological functions of CCNB1 were analyzed through the GeneCards, the Search Tool for the Retrieval of Interacting Genes (STRING), and the Database for Annotation, Visualization, and Integrated Discovery (DAVID) databases. Therapeutic effects of UA on COAD cell lines HCT-116 and SW-480 were analyzed by CCK-8 and high-content screening (HCS) imaging assay. Flow cytometry was utilized to detect cell cycle changes of SW-480 and HCT-116 cells. Levels of mRNA and expression proteins of HCT-116, SW-480, and normal colon epithelial cells NCM-460 were determined by qRT-PCR and western blot.

**Results:** CCNB1 was highly expressed and acted as an oncogene in COAD patients. CCNB1 and its interacting genes were significantly enriched in the cell cycle pathway. UA effectively inhibited the proliferation and injured COAD cells. In addition, UA arrested cell cycle of COAD cells in S phase. With regard to the molecular mechanisms of UA, we demonstrated that UA can significantly downregulate CCNB1 and its interacting genes and proteins, including CDK1, CDC20, CCND1, and CCNA2, which contributed to cell cycle blocking and COAD treatment.

**Conclusion:** Results from this study revealed that UA possesses therapeutic effects on COAD. The anti-COAD activities of UA are tightly related to suppression of CCNB1 and its interacting targets, which is crucial in abnormal cell cycle process.

## Introduction

Colon adenocarcinoma (COAD) is the most commonly diagnosed malignancies among many cancers and also a major causing cancerous death throughout the world. Statistically, the global data showed that newly increased COAD patients approximate 1.1 million and 550 thousand fatalities, and the death rate from COAD is predicted to increase by 60% in 2035 (Bray et al., 2018; Araghi et al., 2019). Furthermore, although breakthrough in early diagnosis and intervention, the survival rates for COAD patients need to be improved. It is noteworthy that COAD patients’ 5-year relative survival rate (10 years after diagnosis) is less than 65% (Siegel et al., 2017). Consequently, it is necessary to seek a new measure for accurate diagnosis and effective treatment of COAD.

Cell cycle includes four successive stages: G1, S, G2, and M phase (Wang and Levin, 2009). Abnormal cell cycle regulation has been recognized as a core engine for the unlimited proliferation of cancer cells. In cancer cells, the mechanisms that protect damaged cells and balance between cellular multiplication and apoptosis are disordered (Huang and Yu, 2015; Kar, 2016). Furthermore, a mushrooming number of research studies demonstrated that cyclin family members conduct a key role in regulating cell cycle progression, and aberrant expression of cyclin would hinder proliferation of tumor cell (Wang et al., 2014; Liu et al., 2015; Xie et al., 2019). Cyclin B1 (CCNB1), a cyclin family member, plays an important role, similar to the switch role, in cell mitosis. CCNB1-encoded proteins are crucial proteins that regulate the G2/M transition phase of the cell cycle (Kong et al., 2000). Besides, CCNB1 has been shown to interact with cyclin-dependent kinase 1 (CDK1) (Shanahan et al., 1999). The complex of CCNB1/CDK1 includes the active complex phosphorylates and activates 13S condensin that promotes some events of early mitosis that it is manifested in condensed chromosomes (Kimura et al., 1998). Furthermore, CCNB1/CDK1 in mitochondria phosphorylated the complex I (CI) subunits and enhanced CI enzymatic activity, subsequently, mitochondrial respiration is increased, and oxygen consumption and ATP generation are increased, providing efficient biological energy for cells, promoting G2/M transformation, and shortening the whole progression of cell cycle (Wang et al., 2014; Xie et al., 2019). Some other cyclin family members, such as cyclin-dependent kinase 4 or cyclin D1 (CCND1), also mediated pathological process in tumor cells and motivated cellular metastasis and tumor invasiveness (Tchakarska et al., 2011; Jin et al., 2015). Obviously, it is logical to infer that cyclins and its catalytic partner, CDKs, are the keys that unlock the mysterious box of cell cycle and develop into a backbone in cancer treatment. Furthermore, it has been reported that inhibition of COAD was associated with reduction of the cell cycle regulator protein CCNB1, which is achieved by upregulating the expression of P21 (Suboj et al., 2012).

Ursolic acid (3β-hydroxy-urs-12-ene-28-oic acid, UA) is a natural pentacyclic triterpenoid carboxylic acid, with a wide range of anticancer activities. A previous study has reported that UA inhibited multiplication, metastasis, and invasiveness of COAD cells *in vitro*. In addition, UA effectively suppressed tumor bulk, angiogenesis, and toward distant organs invasion ability *in vivo* and strengthened the therapeutic effects of capecitabine (Prasad et al., 2012). Another research declared that dietary UA content of 0.11% could reduce the morbidity of abnormal crypt lesions, which is one of the earliest precursors to COAD, especially at the tumor initiation stage (Andersson et al., 2008). Furthermore, UA also blocked cell cycle in G1 stage, causing the increase of cytochrome C in the cytoplasm and caspase-9 and caspase-3 protein in COAD HCT-116 cell lines (Dar et al., 2016). UA can induce apoptosis of SW-480 cells, which may be related to downregulation of Bcl-2, Bcl-XL, and survivin, and UA possesses cytotoxicity on colon cancer cells (Thomadaki and Scorilas, 2008; Shan et al., 2011). Meanwhile, there were several clinical trials (phase I) reported that UA showed antiadvanced solid tumor activities with manageable toxicities (Wang et al., 2013; Zhu et al., 2013; Qian et al., 2015). Therefore, it is evident that UA and its analogues are promising therapeutic agents against COAD.

In the current study, the therapeutic consequence of UA on COAD cells will be investigated *in vitro*. Furthermore, we also explored potential mechanisms of UA in regulating cell cycle, conducing to comprehending superiorly the molecular mechanisms of UA in treating COAD.

## Materials and Methods

### Bioinformatics of CCNB1

In order to explore the expression level, interacting genes, proteins, and signaling pathways of CCNB1, a pivotal regulatory protein in cell cycle, the GeneCards (https://www.genecards.org/) database was used. It provides overall information on predicted human genes with annotations and could be used to search and consolidate data (Safran et al., 2010). The protein-protein interaction (PPI) network of CCNB1 was established by the Search Tool for the Retrieval of Interacting Genes (STRING, http://string-db.org, version 11.0) (Szklarczyk et al., 2015), and the interactional composite score >0.9 was perceived with statistical significance. The visual network of molecular interactions was established by Cytoscape (version 3.7.1, www.cytoscape.org/). The plugin cytoHubba was taken advantage to identify rounding out the top 10 hub genes in PPI based on Maximal Clique Centrality (MCC) methods (Li and Xu, 2019). To further investigate the molecular mechanisms of CCNB1 in COAD, the Database for Annotation, Visualization, and Integrated Discovery database (DAVID, http://david.ncifcrf.gov, version 6.8) was used (Huang et al., 2009). Kyoto Encyclopedia of Genes and Genome (KEGG) enrichment analysis was applied in studying function and cell-signaling paths of CCNB1. Bubble plots of enrichment results were drawn using the “ggplot” package for R software. *p* < 0.05 was considered statistically significant.

The Gene Expression Omnibus (GEO, http://www.ncbi.nlm.nih.gov/geo) database was applied to obtain gene expression profiles. Differentially expressed genes (DEGs) were determined with an empirical Bayesian approach using the Bioconductor “limma” package in R software (Ritchie et al., 2015). For values not reported in logarithmic form, log2 conversion was performed. LogFC (fold change) > 1.5 or logFC < −1.5 and false discovery rate (FDR) < 0.05 were considered as statistically significant. Clinical data and RNA expression level of COAD patients were acquired from The Cancer Genome Atlas (TCGA, https://cancergenome.nih.gov/) database, which included 480 COAD samples and 42 noncancer samples as of October 2020. DEGs were identified between COAD tissue samples and noncancer tissue samples in the TCGA dataset by the Bioconductor “DESeq2” package in R software (version 3.6.0, 64-bit, https://www.r-project.org/) (Love et al., 2014). LogFC >1.5 or logFC < −1.5 and FDR <0.05 regarded as statistical significance.

### Preparation of Ursolic Acid

Criterions of UA (purity 98%, Cat. No. CHB180311) were obtained from Chroma Biotechnology Co. Ltd. (Chengdu, China). When applying to cell lines, UA will be dissolved in dimethyl sulfoxide and diluted to require concentration.

### Cell Lines and Culture

Human-derived COAD cell lines SW-480 (ATA-CL1052) and HCT-116 (CL0125) were purchased from PuJian Cell Center (Wuhan, China) and FengHui Cell Center (Beijing, China), respectively, and human normal colon epithelial cells NCM-460 (ATA-CL1041) were purchased from PuJian Cell Center (Wuhan, China). All cell lines were cultivated in Dulbecco’s Modified Eagle Medium (Gibco, Thermo Fisher Scientific, Inc.) including ten percent fetal bovine serum (Hyclone, GE Healthcare Life Sciences, Logan, UT, United States) and 1% streptomycin and penicillin (Thermo Fisher Scientific, Inc.), then nurtured in 5% CO_2_ at 37 °C. All three types of cells used in the experiment were maintained at 3–5 generations after recovery.

### Cell Viability Evaluation and Morphological Identification

The viability of SW-480 and HCT-116 was processed by UA for 24 h, and 10% (vol/vol) cell counting kit-8 (CCK-8, Lot. PG658, Dojindo, Tokyo, Japan) was added into cells and incubated for 15 min at 37 °C. Absorbance was measured at 450 nm. Cell viability was calculated as cell viability (%) = 100 × (OD treatment/OD control). For SW-480 and HCT-116 cells, the 50% inhibitory concentration (IC_50_) was calculated.

Morphological identification and quantitative statistics of HCT-116 and SW-480 cells were examined via High-Content System (HCS) array scan (Thermo Scientific, Massachusetts, United States). Fluorescent dyes, including Hoechst 33,342 (H3570, Invitrogen) for cell counts quantitatively, Calcein AM (C3099, Invitrogen) for survival cell marking, and ethidium homodimer-1 (EthD-1) (L3224, Invitrogen) for injured cell marking, were employed to identify cells’ morphology. The cell health analysis module was selected in the HCS system, and the fluorescence images were collected by referring to the parameters and methods reported by O'Brien et al., (2006), and the wavelengths of different channels were set. Ultimately, we acquire average fluorescence intensity of HCT-116 and SW-480 cells by using a software algorithm in the ArrayScan XTI system.

### Flow Cytometry (FCM)

Flow cytometry was used to analyze the cell cycle. The cells used for the experiment were collected and washed with cold PBS after that fastened in cold ethanol (70%). Next, propidium iodide (Sigma, United States) was applied to cellular staining for 20 min at room temperature, analyzing cell proportions of each phase by FCM (BD, FACSCanto Ⅱ, United States).

### Real-Time Quantitative PCR for mRNA Expressions

RNA was extracted from SW-480, HCT-116, and NCM-460 cells in microarray analysis to validate the expression level genes in the cell cycle pathway. According to the instructions, TRIzol Reagent (Nordic Bioscience, Beijing, China) was converted to cDNA using a reverse transcription kit (Thermo Scientific, United States). Table 1 exhibits primer sequences of CCNB1, CDK1, CDK2, CCND1, CCNA2 CDC20, CCNB2, and CKS2. cDNA and SYBR Green PCR Master Mix (Nordic Bioscience, Beijing, China) were used to analyze these mRNAs in quantitative real-time PCR. The 7,500 fast real-time PCR system (Applied Biosystems, Foster City, CA, United States) was operated to RT-PCR. β-actin as an endogenous reference to calculate the relative amounts of mRNA.

**TABLE 1:** Primer sequences of real-time PCR analyses for mRNA expression.

Genes	Forward	Reverse
CCNB1	GCC​AGT​GCC​AGA​GCC​AGA​AC	CAT​TGG​GCT​TGG​AGA​GGC​AGT​ATC
CDK1	ACA​GGT​CAA​GTG​GTA​GCC​ATG​A	GCA​TAA​GCA​CAT​CCT​GAA​GAC​TGA​C
CDK2	GCT​CTC​ACT​GGC​ATT​CCT​CTT​CC	GGA​CCC​GAT​GAG​AAT​GGC​AGA​AAG
CCND1	GCC​CTC​GGT​GTC​CTA​CTT​CAA​ATG	TCC​TCC​TCG​CAC​TTC​TGT​TCC​TC
CCNA2	ATG​AGA​GCT​ATC​CTC​GTG​GAC​TGG	GCA​CTG​ACA​TGG​AAG​ACA​GGA​ACC
CDC20	AGC​AGC​AGA​TGA​GAC​CCT​GAG​G	CAG​CGG​ATG​CCT​TGG​TGG​ATG
CCNB2	ACA​ACC​AGA​GCA​GCA​CAA​GTA​GC	AGG​ACC​CTT​TGG​AGC​CAA​CTT​TTC
CKS2	CGA​ACA​CTA​CGA​GTA​CCG​GCA​TG	ACC​AAG​TCT​CCT​CCA​CTC​CTC​TTC
β-Actin	GGC​CAA​CCG​CGA​GAA​GAT​GAC	GGA​TAG​CAC​AGC​CTG​GAT​AGC​AAC

### Western Blotting (WB)

The procedures of WB were performed as previously described by Yang et al. (2020). Details on main antibodies are as follows: rabbit anti-CCNB1 Ab (Proteintech, Cat No. 28603-1-AP, dilution: 1:1,000), rabbit anti-CDC20 Ab (Proteintech, Cat No. 10252-1-AP, dilution: 1:1,000), rabbit anti-CDK1 Ab (Proteintech, Cat No. 19532-1-AP, dilution: 1:1,000), rabbit anti-CCND1 Ab (Proteintech, Cat No. 60186-1-Ig, dilution: 1:1,000), rabbit anti-CCNA2 Ab (Proteintech, Cat No. 18202-1-AP, dilution: 1:1,000), and GAPDH monoclonal antibody (Proteintech, 60004-1-Ig, dilution: 1:10,000). ImageJ Plus software (National Institute of Health, Bethesda, MD, United States) was used to measure in scanned picture of the grey values of blots, and the GAPHD was set as a loading control for the grey value of each target protein.

### Statistics Analysis

The mean standard deviation (SD) is used to show all data by SPSS software (version 20.0, SPSS Inc., Chicago, IL, United States) for analysis. One-way ANOVA and LSD were used to analyze the data. *p* < 0.05 has statistical significance, and *p* < 0.01 was high significantly. GraphPad Prism software for Windows (version 7.0, San Diego, CA, United States) was used as visible display of all results.

## Results

### CCNB1 and Its Interacting Genes Were Significantly Enriched in Cell Cycle Pathway

**FIGURE 1:**
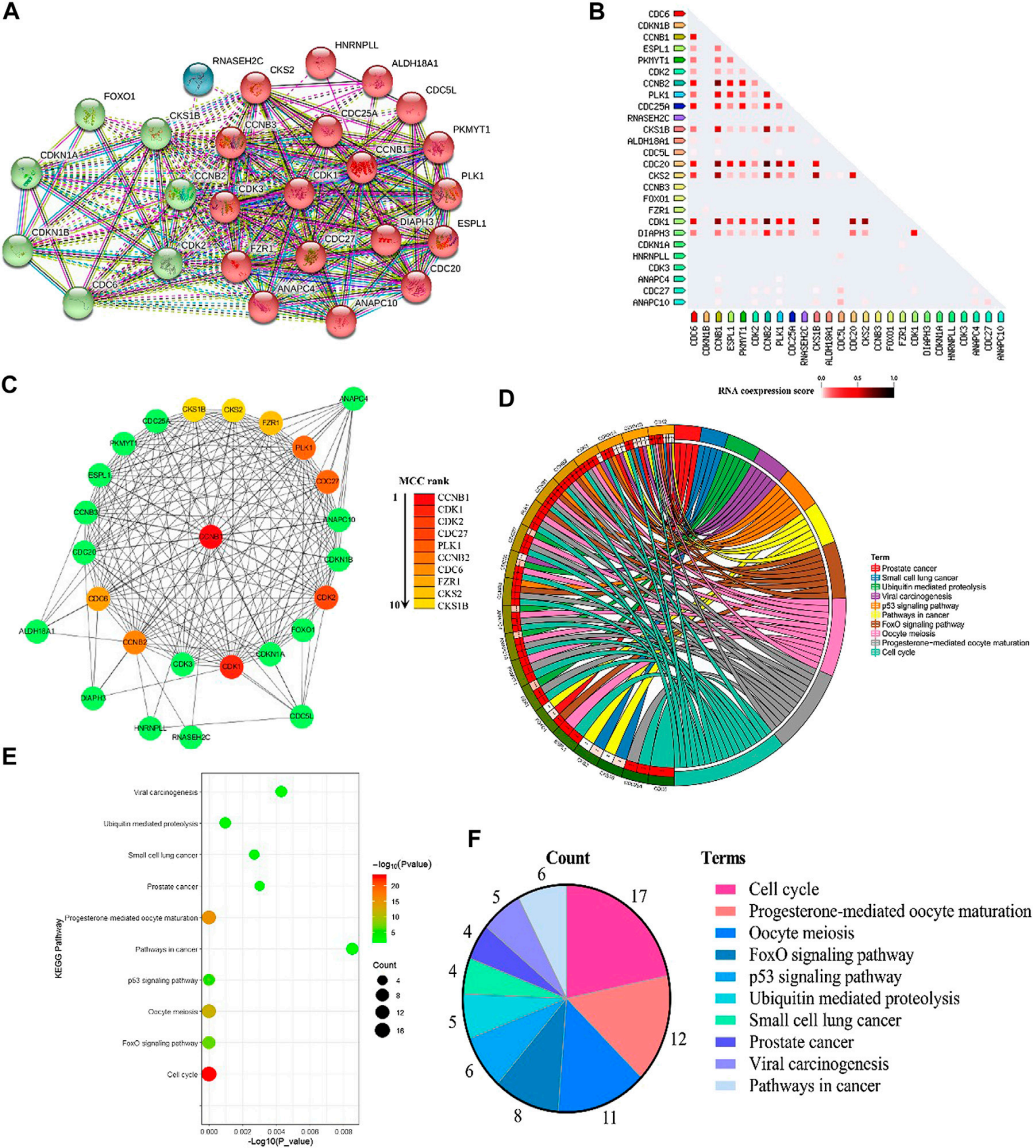
Bioinformatics of CCNB1. **(A)** PPI network of CCNB1; **(B)** the coexpression RNA of CCNB1; **(C)** the top 10 hub genes in the PPI network; **(D)** the top 10 enriched KEGG pathways and enriched genes presented by circus plot; **(E)** the top 10 enriched KEGG pathways presented by bubble plot; **(F)** the top 10 enriched KEGG pathways presented by pie plot.

There were 2,186 interacting genes with CCNB1 in the GeneCards database. Then, the top 25 interacting genes (FZR1, DIAPH3, CDC20, CDC25A, ESPL1, CDC5L, HNRNPLL, PKMYT1, CDC27, ANAPC10, CDK1, CCNB3, CKS2, PLK1, ALDH18A1, CDK3, ANAPC4, CDKN1A, CDK2, CCNB2, CKS1B, CDC6, FOXO1, CDKN1B, and RNASEH2C, respectively), which present the highest relation scores (≥0.95) with CCNB1, were selected. To predict the interactions between interacting proteins and CCNB1, the STRING database was used to establish a PPI network (Figure 1A). In total, CCNB1, FZR1, DIAPH3, CDC20, CDC25A, ESPL1 CDC5L, HNRNPLL, PKMYT1, CDC27, ANAPC10, CDK1, CCNB3, CKS2, PLK1, ALDH18A1, CDK3, and ANAPC4 presented the highest interacting proteins in the PPI network (red nodes). The coexpression RNA of CCNB1 was also explored, and results are presented in Figure 1B. The highest coexpression RNA of CCNB1 was CDK1, CKS2, CDC20, and CCNB2, respectively. Then, the top ten hub genes were identified from Cytoscape software. Consequences are presented in Figure 1C. The top 10 hub genes were CCNB1, CDK1, CDK2, CDC27, PLK1, CCNB2, CDC6, FZR1, CKS2, and CKS1B. The interacting genes of CCNB1 are explored in signaling pathways by KEGG pathway enrichment analysis. The top 10 enriched results are presented in Figures 1D–F. The interacting genes of CCNB1 were significantly enriched in the cell cycle pathway (Figure 1E). In the cell cycle pathway, the enriched genes were CCNB1, CDC6, CDK1, FZR1, ANAPC4, PKMYT1, ANAPC10, CDC20, ESPL1, CDC27, CDC25A, CDK2, CCNB3, CDKN1A, CDKN1B, CCNB2, and PLK1, respectively (Figure 1D).

### CCNB1 Was High Expression in COAD and Connected With Poor Prognosis

**FIGURE 2:**
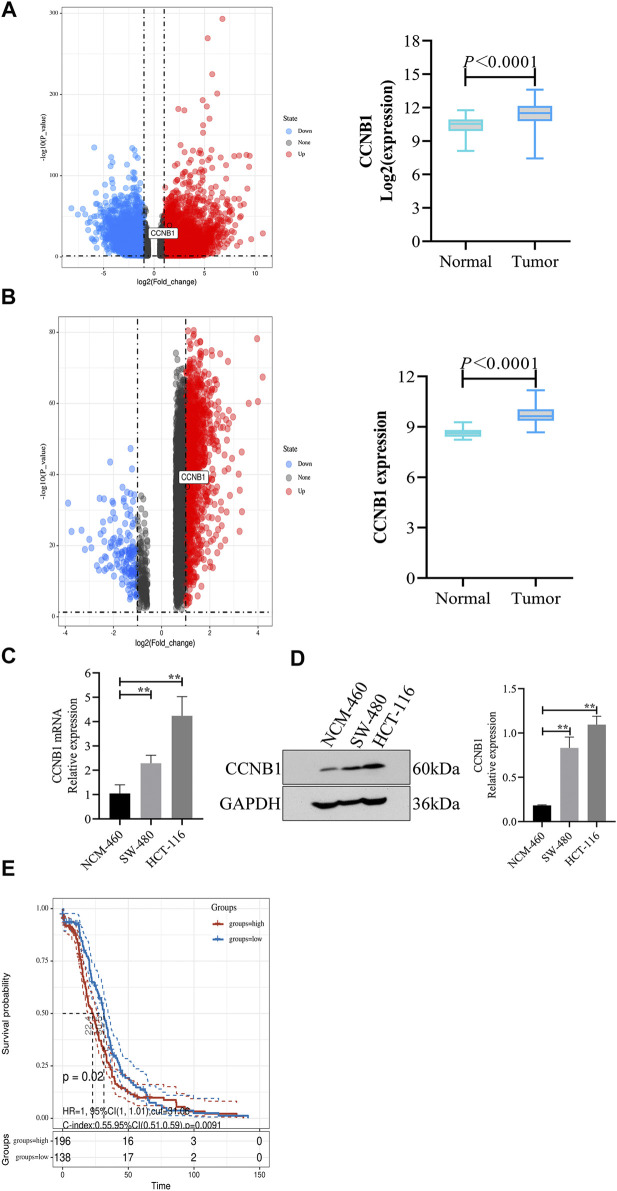
The expression and prognosis of CCNB1 in COAD. **(A)** mRNA expressions of CCNB1 in the TCGA database. Upregulated genes marked as red points, downregulation genes marked as blue points, and no significantly different genes marked as black points. **(B)** mRNA expressions of CCNB1 in the GSE37182. mRNA expressions of CCNB1 in the TCGA database. Upregulated genes marked as red points, downregulation genes marked as blue points, and no significantly different genes marked as black points; **(C)** mRNA expressions of CCNB1 verified by RT-PCR, ∗∗*p* < 0.01 vs. the NCM-460 group. **(D)** Protein expressions of CCNB1 confirmed by WB, ∗∗*p* < 0.01 vs. the NCM-460 group. **(E)** The survival curve of prognostic influence was expressed by CCNB1 in the TCGA database. The red curve represents high expression, and the blue curve represents low expression.

Through analysis of DEG expression in the TCGA database, CCNB1 expression was conspicuously high in tumor samples more than nontumor samples (log FC = 1.53, *p* = 1.25E-39, Figure 2A). Meanwhile, through the analysis of DEG expression in the GEO database, CCNB1 was also highly expressed in tumor tissues (log FC = 1.51, *p* = 2.41E-37, Figure 2B). In addition, the mRNA and protein expressions of CCNB1 in HCT-116 and SW-480 cell lines were significantly higher than those in NCM-460 cells (Figures 2C,D). Then, combined with the clinical information of patients, survival analysis in the TCGA database showed that the total survival time was significantly lower in patients with high CCNB1 expression (*p* = 0.02) (Figure 2E). Therefore, it is logical to assume that CCNB1 considered carcinogen possibly in COAD and was tightly related to poor prognosis.

### UA Inhibited the Proliferation and Injured COAD Cells

**FIGURE 3:**
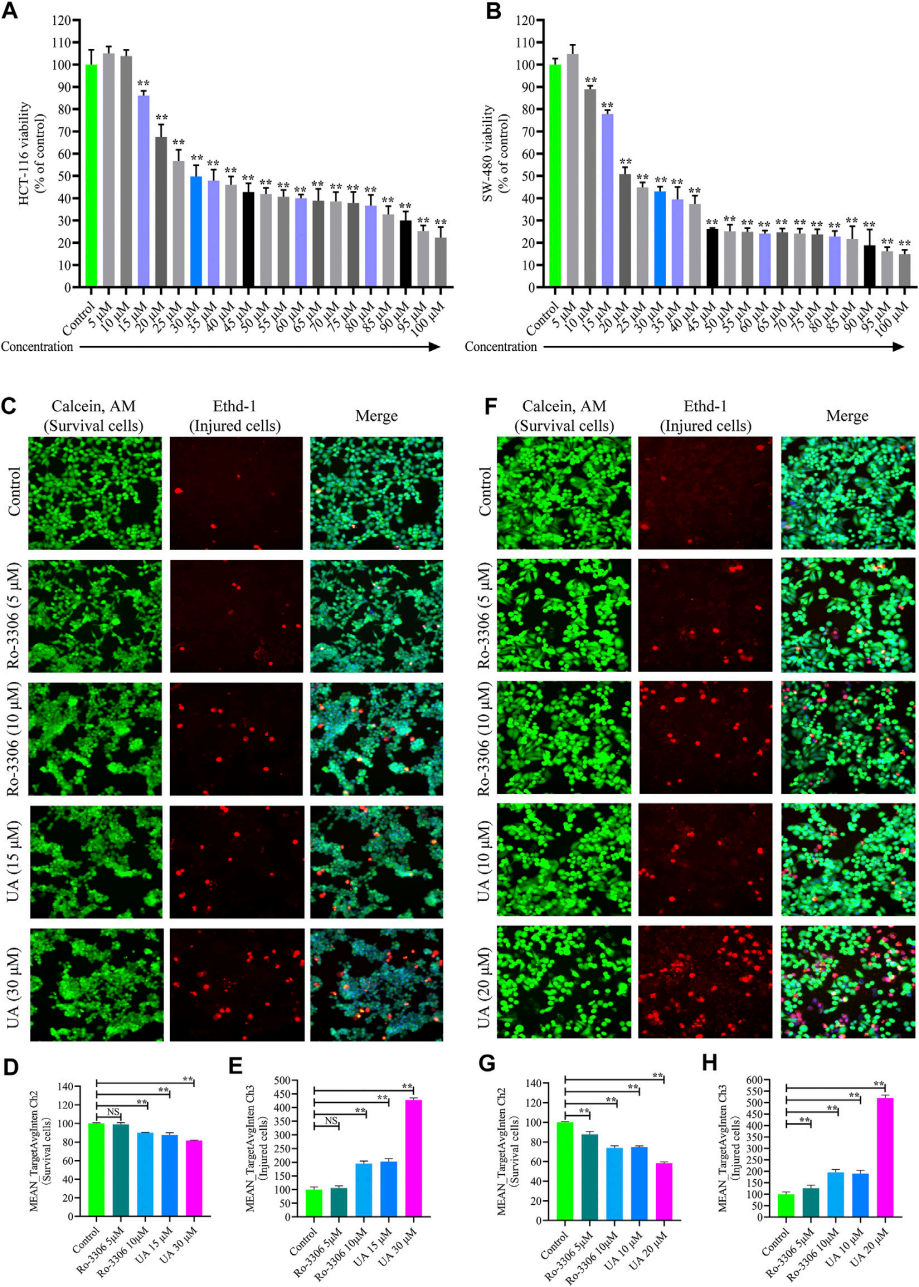
Influences of UA on cell viability and morphology of HCT-116 and SW-480 cells. **(A)** HCT-116 cells in different concentrations of UA (5–100 μM) and the activity of cells; **(B)** SW-480 cells in different concentrations of UA (5–100 μM) and the activity of cells; **(C)** morphological identification of HCT-116 cells. Living cells (green fluorescence) and damaged cells (red fluorescence) were reflected by fluorescence staining intensity. **(D)** Survival cell counts of HCT-116 cells (MEAN_TargetAvgIntenCh2). **(E)** Injured cell counts of HCT-116 cells (MEAN_TargetAvgIntenCh3). **(F)** Morphological identification of SW-480 cells. Living cells (green fluorescence) and damaged cells (red fluorescence) were reflected by fluorescence staining intensity. **(G)** Survival cell counts of SW-480 cells (MEAN_TargetAvgIntenCh2). **(H)** Injured cell counts of SW-480 cells (MEAN_TargetAvgIntenCh3). NS, no significance compared with the control group; ∗∗*p* < 0.01 vs. the control group. The percentage of the control group represents the results, and the mean ± SD represents the data.

The cytoactivity and proliferation of HCT-116 and SW-480 cells were measured by cell counting kit-8 (CCK-8). The IC_50_ concentrations of UA for HCT-116 and SW-480 were explored firstly. The concentrations of UA were set as 5 –100 μM. The experimental results show that UA with 30 μM notably restrained the cytoactivity and proliferation of HCT-116 (49.79 ± 5.03, Figure 3A) cells and with 20 μM notably restrained the cytoactivity and proliferation of SW-480 (50.85 ± 3.02, Figure 3B) cells. Simultaneously, cell viability of HCT-116 with 100 μM concentrations of UA was lower than 75% (22.31 ± 4.70, Figure 3A) and SW-480 was lower than 85% (14.93 ± 1.85, Figure 3B). Accordingly, 30 and 20 μM of UA were set as IC_50_ for HCT-116 and SW-480, respectively.

For the sake of identifying the influences of UA on HCT-116 and SW-480 morphological influence, cell number and morphology were quantitatively determined by HCS cell imaging. The selective small-molecule inhibitor of CCNB1 (Ro-3306, MedChem Express, Shanghai, China) was devoted to block the expression of CCNB1 specifically. As displayed in Figure 3C, HCT-116 cells in the control group and Ro-3306 with 5 μM presented with Calcein, AM, and EthD-1 fluorescence homogeneously. On the contrary, EthD-1 staining showed a significant increase in red fluorescent cells in Ro-3306 with 10 μM and the UA groups (15 and 30 μM). In terms of quantitative statistics, survival cell count of HCT-116 was significantly decreased (Figure 3D) and injured cells increased evidently (Figure 3E) after intervened by Ro-3306 (10 μM) and UA (15 and 30 μM) for 24 h. The same results were reproduced in SW-480 cells. As presented in Figure 3F, in the UA group (10, 20), the green fluorescence intensity of the viable cells was significantly decreased, and the red fluorescence intensity of the damaged cells was significantly increased. Quantitative statistics results also showed a decline in survival cell count (Figure 3G) for SW-480 and a surge of injured cell count (Figure 3H). All data suggested that UA may effectively inhibit the proliferation and injure COAD cells.

### UA Induced Cell Cycle Arrest in S Phase for COAD Cells

**FIGURE 4:**
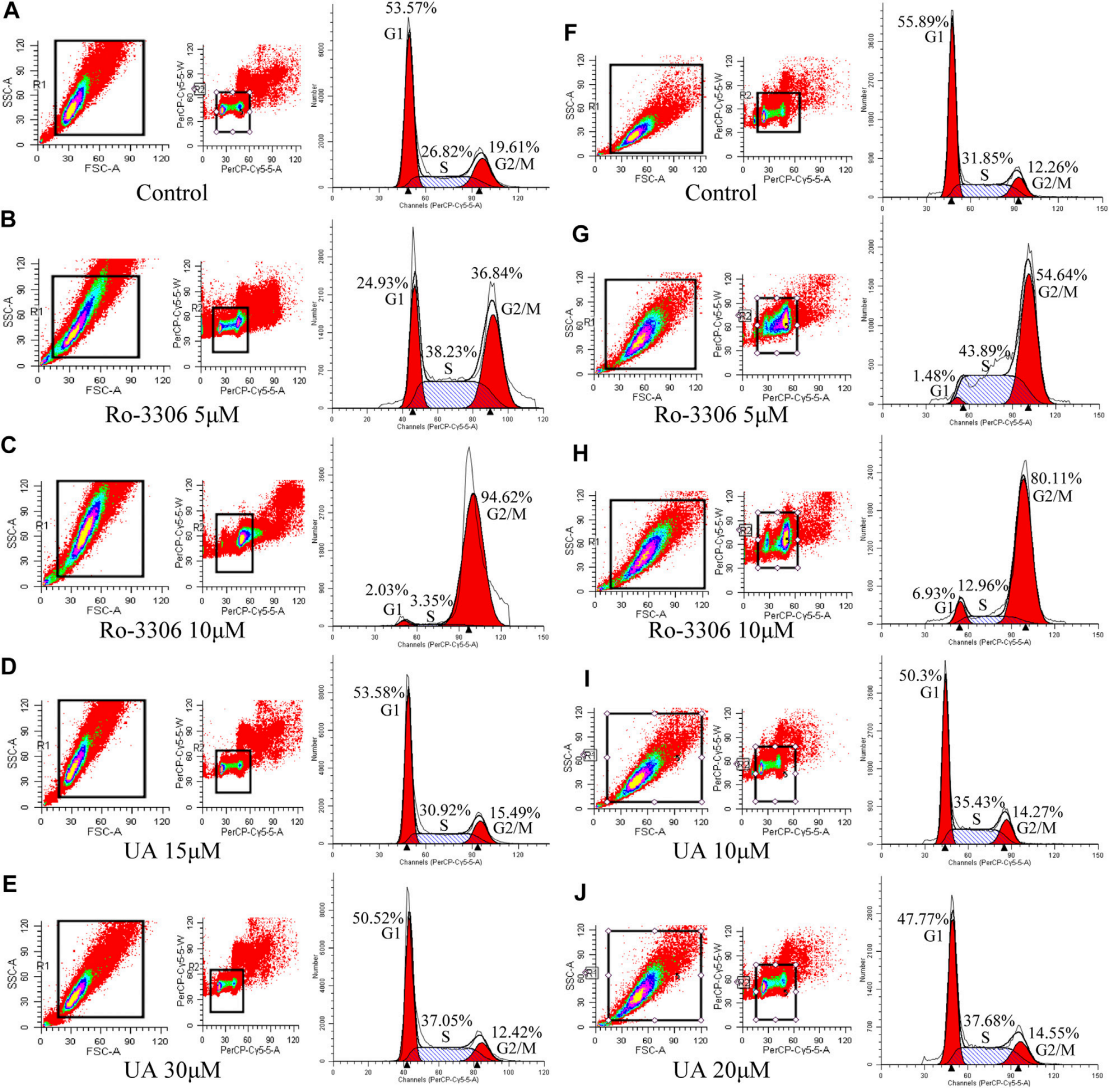
Effects of UA on cell cycle of HCT-116 and SW-480 cells. **(A–E)** The ratio of cells per cell cycle phase of HCT-116 cells followed by Ro-3306 and UA interventions. **(F–J)** The ratio of cells per cell cycle phase of SW-480 cells followed by Ro-3306 and UA interventions.

Cell cycles of HCT-116 and SW-480 were measured by FCM. The results indicate that the proportion of HCT-116 cells in stage S of RO-3306 (5 μM, Figure 4B) and UA (15 and 30 μM) groups (Figure 4D and 4E, respectively) was significantly increased, compared with the control group (Figure 4A), while that in Ro-3306 with 10 μM Figure 4B), the proportion of G2/M phases was significantly higher than other groups. The same results were retrieved in SW-480 cells. Compared with the control group (Figure 4F), the proportion of S phase in SW-480 cells was significantly higher in the Ro-3306 (5 μM, Figure 4G) group and UA (10 and 20 μM) group (Figures 4I,J, respectively), while the proportion of G2/M phases was significantly higher in the Ro-3306 with 10 μM group (Figure 4H). All data manifested that UA induced cell cycle arrest in S phase and characterized with dose-dependent.

### UA Downregulated CCNB1 and Its Interacting Gene Expression

**FIGURE 5:**
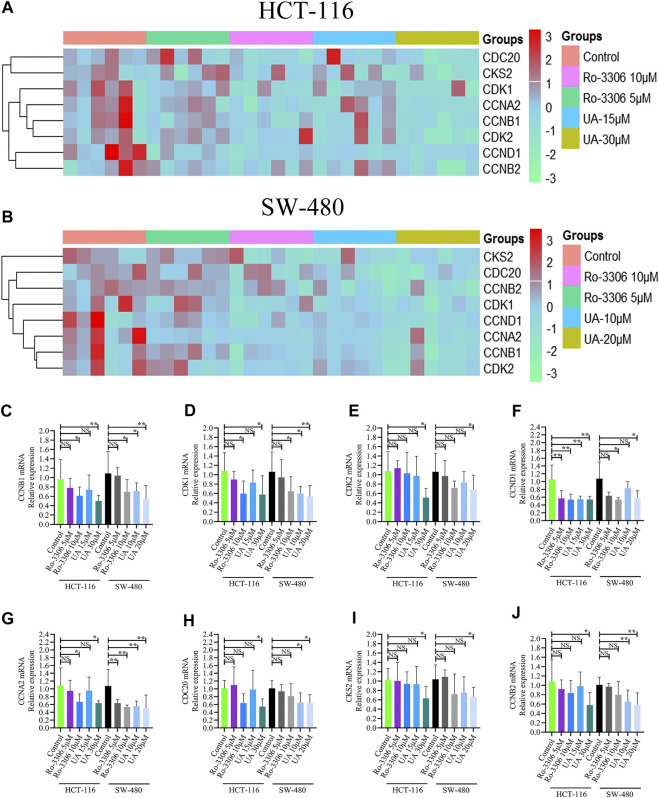
Influences of UA on the mRNA expressions in HCT-116 and SW-480 cells. NS, there was no significance compared with control group; ∗*p* < 0.05 vs. the control group; ∗∗*p* < 0.01 vs. the control group. **(A)** Heatmap of mRNA expression for HCT-116 cells; **(B)** heatmap of mRNA expression for SW-480 cells; **(C)** mRNA expression of CCNB1; **(D)** mRNA expression of CDK1; **(E)** mRNA expression of CDK2; **(F)** mRNA expression of CCND1; **(G)** mRNA expression of CCNA2; **(H)** mRNA expression of CDC20; **(I)** mRNA expression of CKS2; **(J)** mRNA expression of CCNB2. CCNB1, cyclin B1; CDK1, cyclin-dependent kinase 1; CDK2, cyclin-dependent kinase 2; CCND1, cyclin D1; CCNA2, cyclin A2; CDC20, cell division cycle 20; CKS2, CDC28 protein kinase regulatory subunit 2; CCNB2, cyclin B2.

The influence of UA on expression of CCNB1 and its interacting genes in the cell cycle pathway was determined by RT-PCR. Results are presented in Figures 5A,B with heatmap. Evidently, the mRNA expression of CCNB1 and its interacting genes, including CDK1, CDK2, CDC20, CKS2, CCND1, CCNA2, and CCNB2, in HCT-116 and SW-480 cells was decreased significantly compared with the control group. As presented in Figure 5C, the mRNA expression of CCNB1 was significantly suppressed by Ro-3306 and UA interventions, especially in UA with 30 μM for HCT-116 cells and 20 μM for SW-480 cells groups. In addition, the interacting genes of CCNB1, which were significantly enriched in the cell cycle pathway, including CDK1 (Figure 5D), CDK2 (Figure 5E), CCND1 (Figure 5F), CCNA2 (Figure 5G), CDC20 (Figure 5H), CKS2 (Figure 5I), and CCNB2 (Figure 5J), were also downregulated significantly in HCT-116 and SW-480 cells. Consequently, those results mentioned above revealed that UA performs its tumor-inhibitory function by downregulating genes related to CCNB1 and arresting the cell cycle of HCT-116 and SW-480 cells.

### UA Downregulated CCNB1 and Its Interacting Protein Expression

**FIGURE 6:**
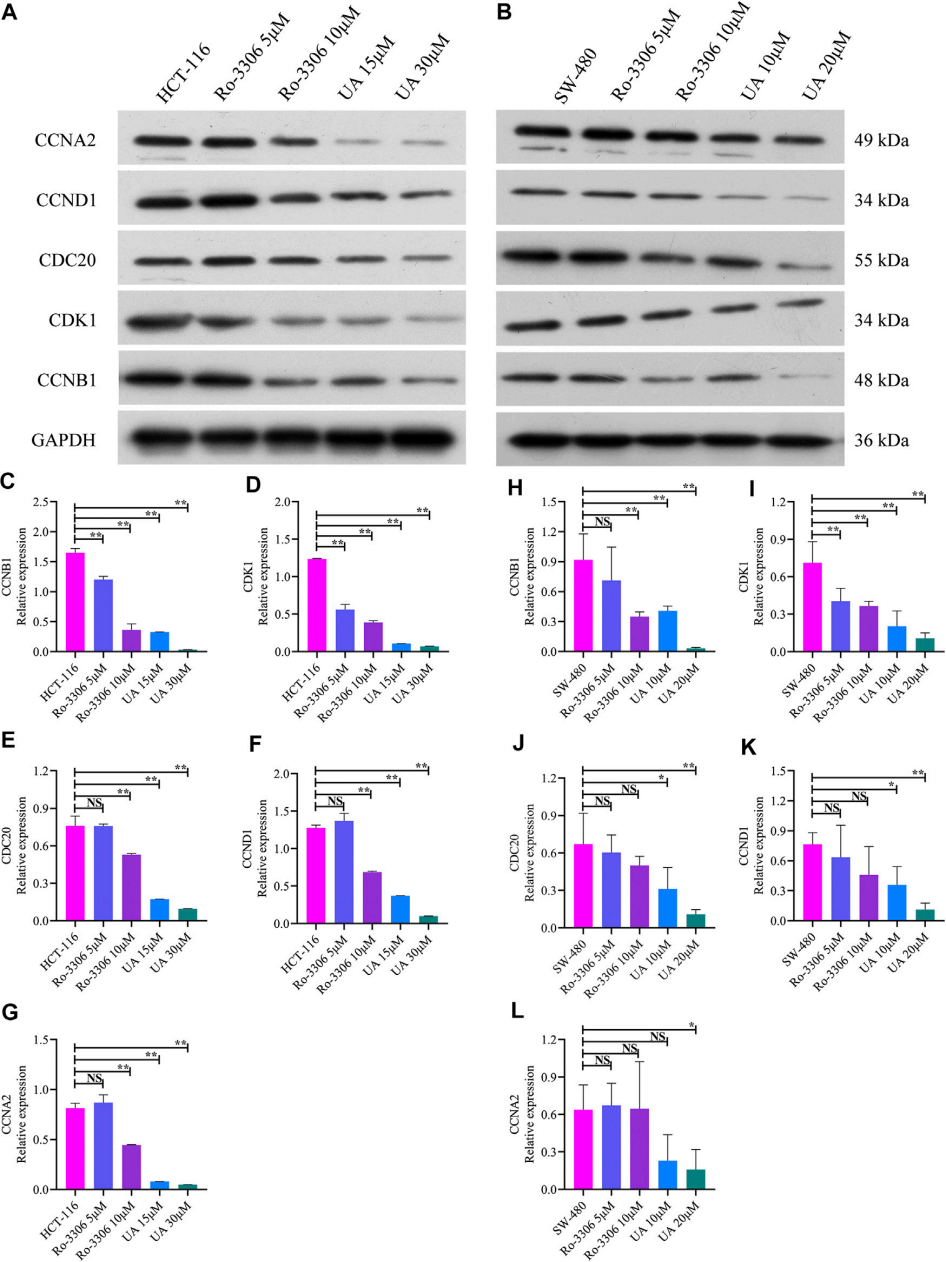
Protein expression of CCNB1 and its interacting proteins analyzed by western blotting analysis. **(A)** Western blotting images of CCNB1, CDK1, CDC20, CCND1, and CCNA2 in HCT-116 cells. **(B)** Western blotting images of CCNB1, CDK1, CDC20, CCND1, and CCNA2 in SW-480 cells. **(C)** Relative CCNB1 protein level in HCT-116 cells. **(D)** Relative CDK1 protein level in HCT-116 cells. **(E)** Relative CDC20 protein level in HCT-116 cells. **(F)** Relative CCND1 protein level in HCT-116 cells. **(G)** Relative CCNA2 protein level in HCT-116 cells. **(H)** Relative CCNB1 protein level in SW-480 cells. **(I)** Relative CDK1 protein level in SW-480 cells. **(J)** Relative CDC20 protein level in SW-480 cells. **(K)** Relative CCND1 protein level in SW-480 cells. **(L)** Relative CCNA2 protein level in SW-480 cells. NS, nonsignificant vs. HCT-116 and SW-480 groups; *p < 0.05 vs. HCT-116 and SW-480 groups; **p < 0.01 vs. HCT-116 and SW-480 groups. CCNB1, cyclin B1; CDK1, cyclin-dependent kinase 1; CDC20, cell division cycle 20; CCND1, cyclin D1; CCNA2, cyclin A2.

As mentioned earlier, the mRNA levels of CCNB1 and its interacting genes in the cell cycle pathway obviously decrease after UA intervention. Then, the protein expression relevant to CCNB1 was explored. Consistent with the results of mRNA, CCNB1, CDK1, CDC20, CCND1, and CCNA2 expressed at a relatively high level in HCT-116 and SW-480 cells (Figures 6A,B). However, when CCNB1 was inhibited by Ro-3306, the protein expressions of CCNB1 and its interacting proteins, including CDK1, CDC20, CCND1, and CCNA2, were downregulated significantly in HCT-116 and SW-480 cells (Figures 6A,B). Remarkably, the expressions of these proteins were significantly suppressed by UA interventions, especially in UA with 30 μM for HCT-116 cells (Figures 6C–G) and 20 μM for SW-480 cells groups (Figures 6H–6L). In addition, the protein expressions of CCNB1, CDK1, CDC20, CCND1, and CCNA2 were exceedingly and significantly different in the low-dose group (15 μM for HCT-116, 10 μM for SW-480) and high-dose group (30 μM for HCT-116, 20 μM for SW-480). Findings from WB analysis provided stronger convincement that UA is able to arrest cell cycle of COAD cells through CCNB1 and its interacting proteins suppressing.

## Discussion

Cell cycle dysregulation is one of the characteristics of COAD. Abnormal cell cycle regulation has been a critical inducement of the imbalance between cell multiplication and apoptosis, also preventing cell damage (Huang and Yu, 2015; Kar, 2016; Xie et al., 2019). CCNB1, a core regulatory protein involved in mitosis and cell cycle, was explored in this research. The significant upregulation of CCNB1 in COAD was confirmed in this study. In addition, results from this study revealed that UA, a natural pentacyclic triterpenoid carboxylic acid, could arrest cell cycle of COAD cells in S phase and downregulate mRNA, as well as protein expression of CCNB1.

Cell transition from G2 phase to M phase is the normal operation of CCNB1, but overexpression of CCNB1 can lead to uncontrolled cell growth and carcinogenesis by binding to its partner CDKs (such as CDK1) (Wang et al., 2014; Xie et al., 2019). Unregulated phosphorylation and proliferation of other substrates at inappropriate times may be associated with CDK binding (Yuan et al., 2006). Previous studies have confirmed that CCNB1 is highly expressed in multiple cancers, including cervical cancer, gastric cancer, head, and neck squamous cell carcinoma, non-small-cell lung cancer, prostate cancer, oral cancer, and esophageal cancer (Zhang et al., 2018; Farshadi et al., 2019; Żuryń et al., 2019). The chromosomal instability and invasiveness of breast cancer might be caused by the high expression of CCNB1, which usually precedes immobilization and aneuploidy of tumor cells (Suzuki et al., 2007). CCNB1 is significantly correlated with the degree of tumor infiltration, aggressiveness, and adverse clinical outcome of the patients (Fang et al., 2014; Fei et al., 2019). In addition, due to interacting with CDK1, the complex of CCNB1/CDK1 accelerates certain occurrences of early mitosis (Kimura et al., 1998). Furthermore, CCNB1/CDK1 in mitochondria phosphorylated the complex I (CI) subunits and enhanced CI enzymatic activity, subsequently, mitochondrial respiration is increased, and oxygen consumption and ATP generation are increased, providing efficient biological energy for cells, promoting G2/M transformation, and shortening the whole progression of cell cycle (Wang et al., 2014; Xie et al., 2019). Cell cycle is a continuous system of biological processes, and several studies have elucidated that the lower level of CDK1 and CCNB1 in colon tumor cells slowed down the G2/M phase of the cell cycle, which owned to the retardation of cell cycle in the S phase (Ryu et al., 2009; Wang et al., 2017). We inferred that the S phase arrest in cell cycle could prevent cancer cells entering the G2/M phase. In addition, short-term follow-up of COAD patients revealed that CCNB1 may be a potential biomarker for poor prognosis. There is no doubt that CCNB1 and its interacting target suppression will be a novel drug target in clinical practice (Ingham and Schwartz, 2017). UA, a natural pentacyclic triterpenoid carboxylic acid, extracted from various traditional Chinese medicines, could be a promising therapeutic agent against COAD. Clinical trials have reported UA with antiadvanced solid tumor activities and with manageable toxicities (Wang et al., 2013; Zhu et al., 2013; Qian et al., 2015).

In this current context, we explored the mRNA and protein expression of CCNB1 firstly, and results showed that CCNB1 was significantly upregulated in clinical datasets and COAD cells. Meanwhile, we revealed that CCNB1 was significantly enriched in the cell cycle pathway and interacting with CDK1, CDK2, CDC27, PLK1, CCNB2, CDC6, FZR1, CKS2, and CKS1B via bioinformatics analysis. CDK1, CKS2, CDC20, and CCNB2 were identified as the highest coexpression RNA of CCNB1. All these results indicated that the high expression levels of CCNB1 and its interacting genes or proteins may contribute to abnormal cell cycle regulation. Then, the pharmacological effects and potential mechanisms of UA in cell cycle regulation were explored. Findings from this research revealed that UA was capable of downregulating the expression of CCNB1 and arresting cell cycle of COAD cells (HCT-116 and SW-480) in S phase. As described previously, S phase in cell cycle was for DNA replication (Wang and Levin, 2009). S phase blocking can lead to a DNA-damage response and cell cycle arrest prior to mitosis (Adeyemi et al., 2010; Adeyemi and Pintel, 2014). We found a surge of injured COAD cells and proliferation inhibition followed by UA and the specific inhibitor of CCNB1 intervention. All data suggested that UA could effectively inhibit the proliferation and injure COAD cells, as well as arrest cell cycle in S phase through CCNB1 suppressing. In addition, the interacting genes and proteins, including CDK1, CDC20, CCND1, and CCNA2, were downregulated significantly in HCT-116 and SW-480 cells under the intervention of UA. Therefore, we confirmed that the potential mechanisms of UA in regulating cell cycle of COAD were tightly related to CCNB1 and its interacting gene and protein suppression.

All in all, conclusions from this study suggest that CCNB1 is an oncogene in COAD and is tightly associated with poor prognosis. UA can effectively inhibit the proliferation of COAD and arrest cell cycle of COAD cells in S phase by downregulating CCNB1 and its interacting genes and proteins. Hence, it could conceivably be hypothesized that UA will develop into a promising therapeutic agent against COAD, and this would be a new direction for our future research.

## Data Availability

The datasets presented in this study can be found in online repositories. The names of the repository/repositories and accession numbers can be found in the article/Supplementary Material.
