# Supplementary figures and images for: Ursolic Acid Regulates Cell Cycle and Proliferation in Colon Adenocarcinoma by Suppressing Cyclin B1
Source: Front Pharmacol. 2021 Jan 19;11:622212. doi: 10.3389/fphar.2020.622212 (PMC7898669; doi:10.3389/fphar.2020.622212)

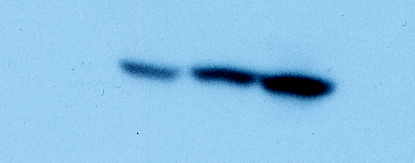

Supplement: Supplementary file 1 [file datasheet1.zip › Original Western blotting images/CCNB1-1.tif]

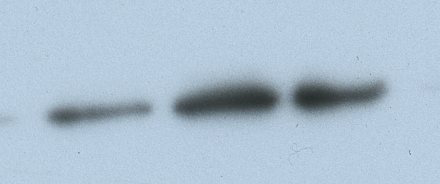

Supplement: Supplementary file 1 [file datasheet1.zip › Original Western blotting images/CCNB1-2.tif]

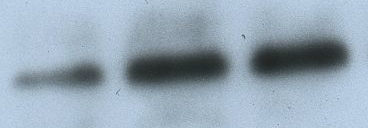

Supplement: Supplementary file 1 [file datasheet1.zip › Original Western blotting images/CCNB1-3.tif]

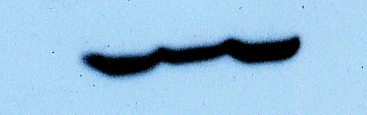

Supplement: Supplementary file 1 [file datasheet1.zip › Original Western blotting images/GAPDH-1.tif]

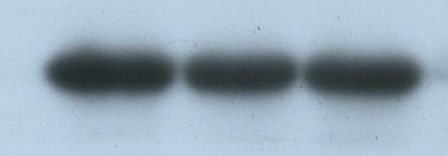

Supplement: Supplementary file 1 [file datasheet1.zip › Original Western blotting images/GAPDH-2.tif]

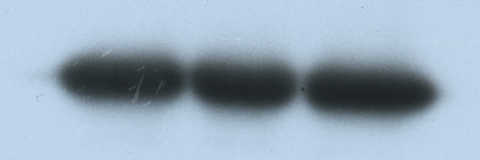

Supplement: Supplementary file 1 [file datasheet1.zip › Original Western blotting images/GAPDH-3.tif]

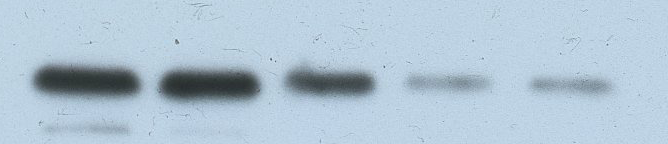

Supplement: Supplementary file 1 [file datasheet1.zip › Original Western blotting images/HCT116-CCNA2-1.tif]

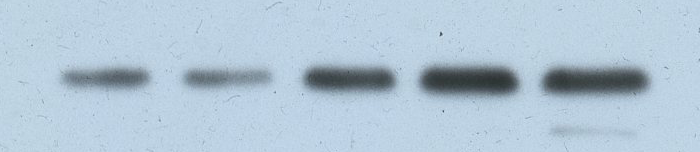

Supplement: Supplementary file 1 [file datasheet1.zip › Original Western blotting images/HCT116-CCNA2-2.tif]

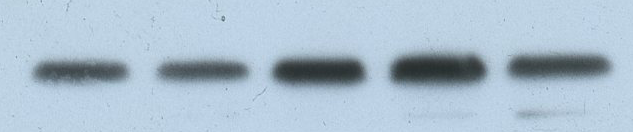

Supplement: Supplementary file 1 [file datasheet1.zip › Original Western blotting images/HCT116-CCNA2-3.tif]

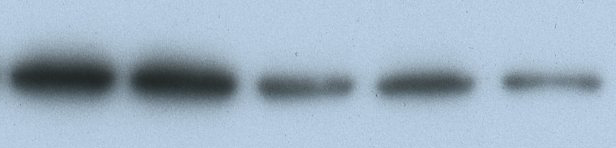

Supplement: Supplementary file 1 [file datasheet1.zip › Original Western blotting images/HCT116-CCNB1-1.tif]

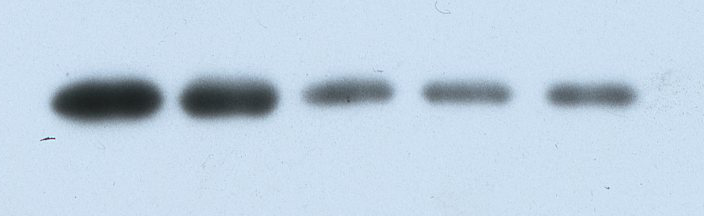

Supplement: Supplementary file 1 [file datasheet1.zip › Original Western blotting images/HCT116-CCNB1-2.tif]

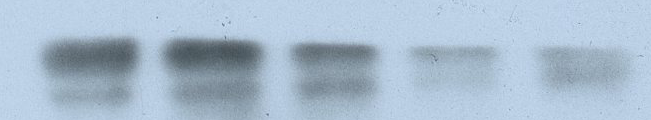

Supplement: Supplementary file 1 [file datasheet1.zip › Original Western blotting images/HCT116-CCNB1-3.tif]

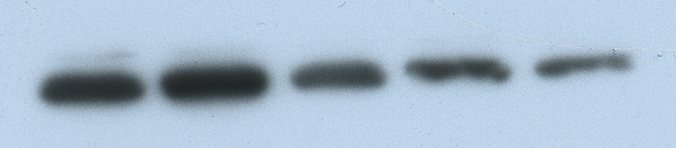

Supplement: Supplementary file 1 [file datasheet1.zip › Original Western blotting images/HCT116-CCND1-1.tif]

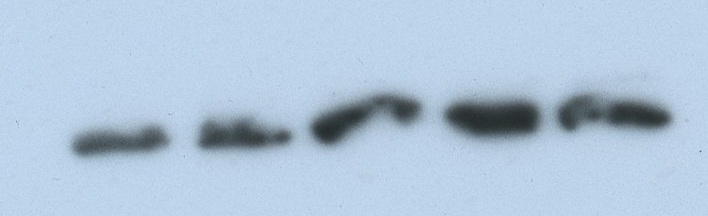

Supplement: Supplementary file 1 [file datasheet1.zip › Original Western blotting images/HCT116-CCND1-2.tif]

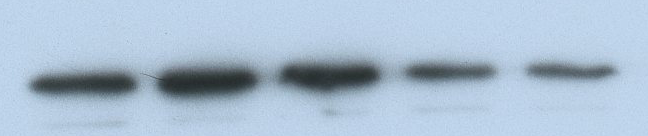

Supplement: Supplementary file 1 [file datasheet1.zip › Original Western blotting images/HCT116-CCND1-3.tif]

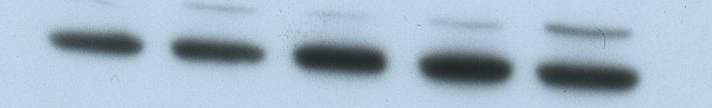

Supplement: Supplementary file 1 [file datasheet1.zip › Original Western blotting images/HCT116-CDC20-1.tif]

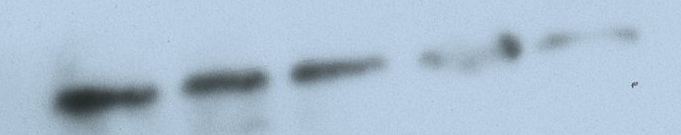

Supplement: Supplementary file 1 [file datasheet1.zip › Original Western blotting images/HCT116-CDC20-2.tif]

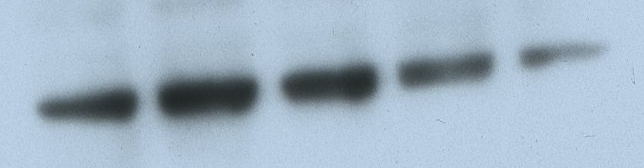

Supplement: Supplementary file 1 [file datasheet1.zip › Original Western blotting images/HCT116-CDC20-3.tif]

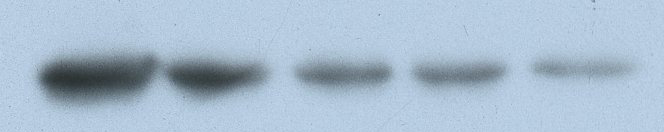

Supplement: Supplementary file 1 [file datasheet1.zip › Original Western blotting images/HCT116-CDK1-1.tif]

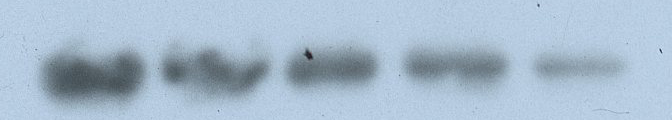

Supplement: Supplementary file 1 [file datasheet1.zip › Original Western blotting images/HCT116-CDK1-2.tif]

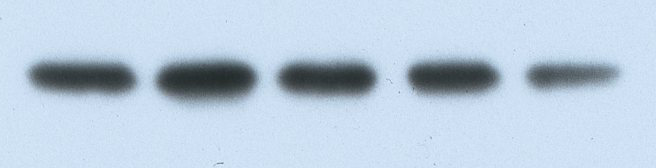

Supplement: Supplementary file 1 [file datasheet1.zip › Original Western blotting images/HCT116-CDK1-3.tif]

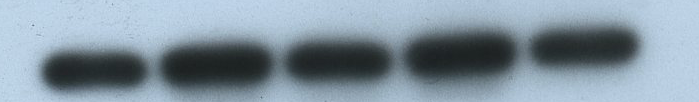

Supplement: Supplementary file 1 [file datasheet1.zip › Original Western blotting images/HCT116-GAPDH-1.tif]

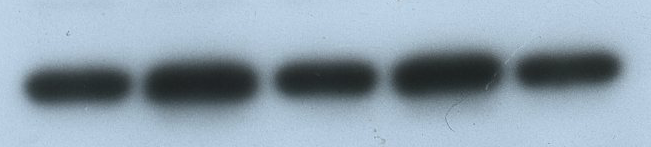

Supplement: Supplementary file 1 [file datasheet1.zip › Original Western blotting images/HCT116-GAPDH-2.tif]

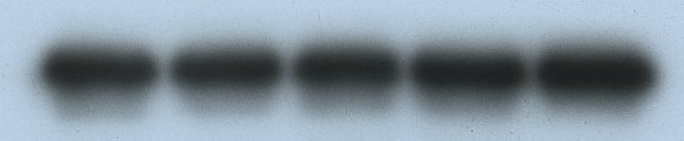

Supplement: Supplementary file 1 [file datasheet1.zip › Original Western blotting images/HCT116-GAPDH-3.tif]

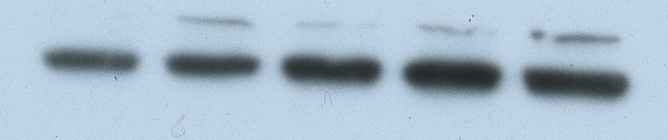

Supplement: Supplementary file 1 [file datasheet1.zip › Original Western blotting images/SW480-CCNA2-1.tif]

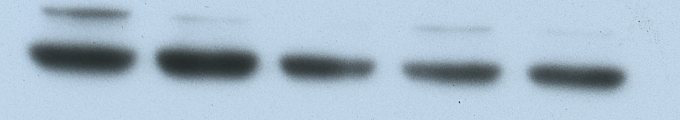

Supplement: Supplementary file 1 [file datasheet1.zip › Original Western blotting images/SW480-CCNA2-2.tif]

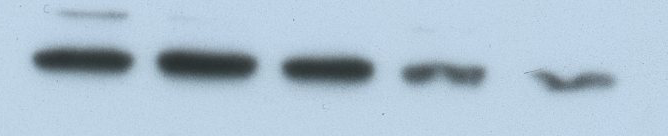

Supplement: Supplementary file 1 [file datasheet1.zip › Original Western blotting images/SW480-CCNA2-3.tif]

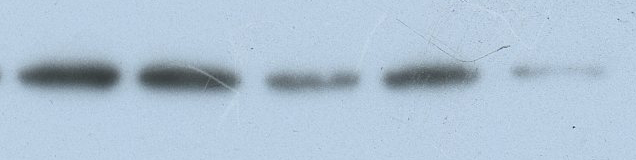

Supplement: Supplementary file 1 [file datasheet1.zip › Original Western blotting images/SW480-CCNB1-1.tif]

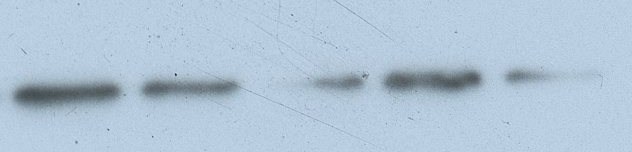

Supplement: Supplementary file 1 [file datasheet1.zip › Original Western blotting images/SW480-CCNB1-2.tif]

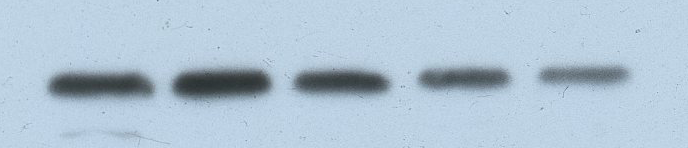

Supplement: Supplementary file 1 [file datasheet1.zip › Original Western blotting images/SW480-CCNB1-3.tif]

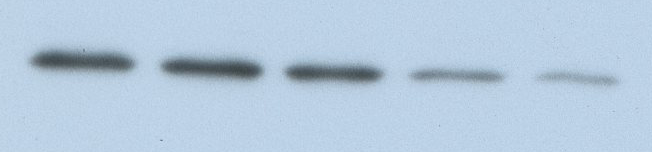

Supplement: Supplementary file 1 [file datasheet1.zip › Original Western blotting images/SW480-CCND1-1.tif]

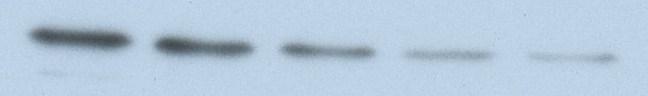

Supplement: Supplementary file 1 [file datasheet1.zip › Original Western blotting images/SW480-CCND1-2.tif]

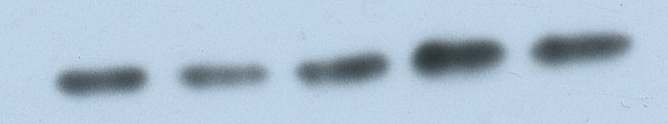

Supplement: Supplementary file 1 [file datasheet1.zip › Original Western blotting images/SW480-CCND1-3.tif]

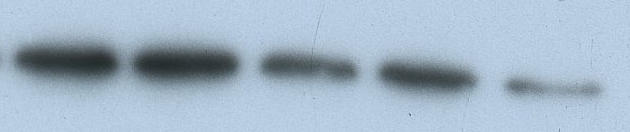

Supplement: Supplementary file 1 [file datasheet1.zip › Original Western blotting images/SW480-CDC20-1.tif]

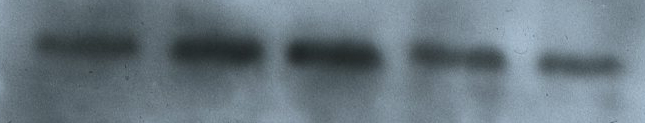

Supplement: Supplementary file 1 [file datasheet1.zip › Original Western blotting images/SW480-CDC20-2.tif]

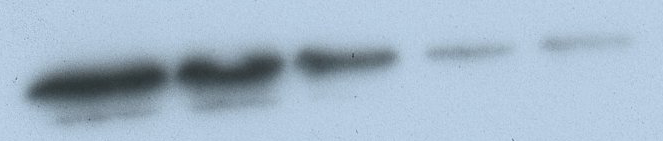

Supplement: Supplementary file 1 [file datasheet1.zip › Original Western blotting images/SW480-CDC20-3.tif]

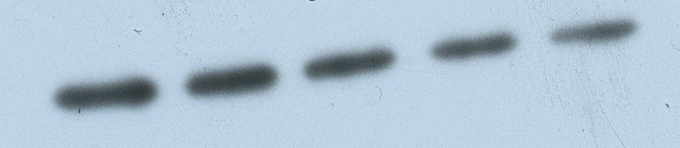

Supplement: Supplementary file 1 [file datasheet1.zip › Original Western blotting images/SW480-CDK1-1.tif]

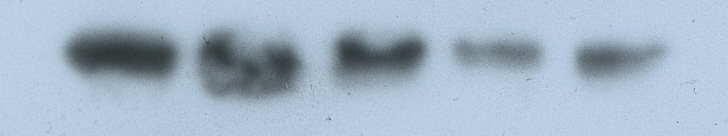

Supplement: Supplementary file 1 [file datasheet1.zip › Original Western blotting images/SW480-CDK1-2.tif]

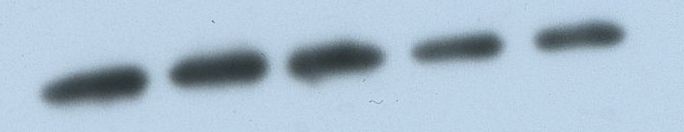

Supplement: Supplementary file 1 [file datasheet1.zip › Original Western blotting images/SW480-CDK1-3.tif]

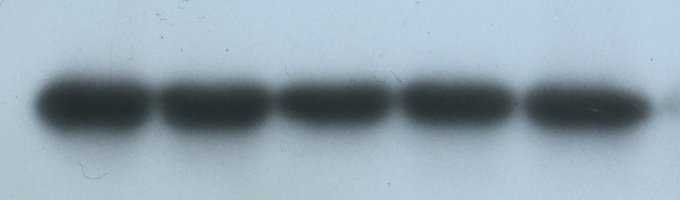

Supplement: Supplementary file 1 [file datasheet1.zip › Original Western blotting images/SW480-GAPDH-1.tif]

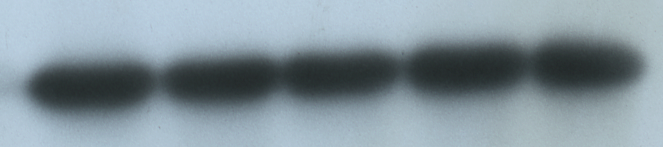

Supplement: Supplementary file 1 [file datasheet1.zip › Original Western blotting images/SW480-GAPDH-2.tif]

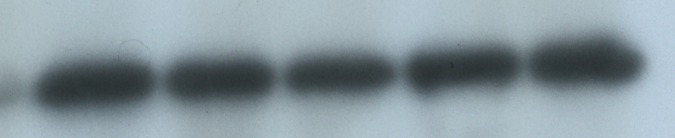

Supplement: Supplementary file 1 [file datasheet1.zip › Original Western blotting images/SW480-GAPDH-3.tif]
